# Supplementary material for: Age at identification, prevalence and general health of children with autism: observational study of a whole country population
Source: BMJ Open. 2019 Jul 9;9(7):e025904. doi: 10.1136/bmjopen-2018-025904 (PMC6629388; doi:10.1136/bmjopen-2018-025904)
Supplement: Author's manuscript [file bmjopen-2018-025904.draft_revisions.pdf]

BMJ Open is committed to open peer review. As part of this commitment we make the peer review history of every article we publish publicly available.

When an article is published we post the peer reviewers' comments and the authors' responses online. We also post the versions of the paper that were used during peer review. These are the versions that the peer review comments apply to.

The versions of the paper that follow are the versions that were submitted during the peer review process. They are not the versions of record or the final published versions. They should not be cited or distributed as the published version of this manuscript.

BMJ Open is an open access journal and the full, final, typeset and author-corrected version of record of the manuscript is available on our site with no access controls, subscription charges or pay-per-view fees (http://bmjopen.bmj.com).

If you have any questions on BMJ Open's open peer review process please email info.bmjopen@bmj.com

BMJ Open

Age at identification, prevalence, and general health of children with autism - observational study of a whole country population

Journal:	*BMJ Open*
Manuscript ID	bmjopen-2018-025904
Article Type:	Research
Date Submitted by the Author:	07-Aug-2018

Complete List of Authors:	Rydzewska, Ewelina; University of Glasgow Mental Health and Wellbeing, Institute of Health and Wellbeing<br>Hughes-McCormack, Laura; University of Glasgow Mental Health and Wellbeing, Institute of Health and Wellbeing<br>Gillberg, Christopher; The University of Glasgow, Institute of Health and Wellbeing, College of Medical, Veterinary and Life Sciences<br>Henderson, Angela; University of Glasgow Mental Health and Wellbeing, Institute of Health and Wellbeing<br>MacIntyre, Cecilia; National Records of Scotland<br>Rintoul, Julie; Scottish Government, Health & Social Care Analysis<br>Cooper, Sally-Ann; University of Glasgow, Medicine

Keywords:	autism, general health, children, young people, prevalence, PUBLIC HEALTH

SCHOLARONE™
Manuscripts

For peer review only

Age at identification, prevalence, and general health of children with autism - observational study of a whole country population

Ewelina Rydzewska, Laura A Hughes-McCormack, Christopher Gillberg, Angela Henderson, Cecilia MacIntyre, Julie Rintoul, Sally-Ann Cooper

Institute of Health and Wellbeing, University of Glasgow, Mental Health and Wellbeing Group, 1st Floor, Administrative Building, Gartnavel Royal Hospital, 1055 Great Western Road, Glasgow G12 0XH, UK
Ewelina Rydzewska
Research Associate

Institute of Health and Wellbeing, University of Glasgow, Mental Health and Wellbeing Group, 1st Floor, Administrative Building, Gartnavel Royal Hospital, 1055 Great Western Road, Glasgow G12 0XH, UK
Laura A Hughes-McCormack
Research Assistant

1 Göteborgs Universitet/University of Gothenburg, Gillbergcentrum/Gillberg Neuropsychiatry Centre, Kungsgatan 12, S-411 19 Göteborg, Sweden
2 Institute of Health and Wellbeing, University of Glasgow, Mental Health and Wellbeing Group, 1st Floor, Administrative Building, Gartnavel Royal Hospital, 1055 Great Western Road, Glasgow G12 0XH, UK
Christopher Gillberg
Professor of Child and Adolescent Psychiatry

Institute of Health and Wellbeing, University of Glasgow, Mental Health and Wellbeing Group, 1st Floor, Administrative Building, Gartnavel Royal Hospital, 1055 Great Western Road, Glasgow G12 0XH, UK
Angela Henderson
Scottish Learning Disabilities Observatory Deputy Director

Census User Needs, Content and Benefits, National Records of Scotland, Ladywell House, Edinburgh EH12 7TF, UK
Cecilia MacIntyre
Statistician

Health & Social Care Analysis, Scottish Government, St. Andrew's House, Regent Road, Edinburgh EH1 3DG, UK
Julie Rintoul
Statistician

Institute of Health and Wellbeing, University of Glasgow, Mental Health and Wellbeing Group, 1st Floor, Administrative Building, Gartnavel Royal Hospital, 1055 Great Western Road, Glasgow G12 0XH, UK
Sally-Ann Cooper
Professor of Learning Disabilities

Corresponding author:
Professor Sally-Ann Cooper
Email: Sally-Ann.Cooper@glasgow.ac.uk

For peer review only - http://bmjopen.bmj.com/site/about/guidelines.xhtml

Abstract

Objectives:

Reported childhood prevalence of autism varies considerably between studies and over time, and general health status has been little investigated. We aimed to investigate contemporary prevalence of reported autism by age, and general health status of children/young people with and without autism.

Design:

Secondary analysis of Scotland's Census, 2011 data. Cross-sectional study.

Setting:

General population of Scotland.

Participants:

All children (n=916,331) and young people (n=632,488) in Scotland.

Main outcome measures:

Number (%) of children/young people reported to have autism; and their general health status. Prevalence of autism; prevalence of poor health (fair, bad and very bad health); odds ratios (OR: 95% confidence intervals) of autism predicting poor health, adjusted for age and gender; and OR for age and gender in predicting poor health within the population with reported autism.

Results:

Autism was reported for 17,348/916,331 (1.9%) children aged 0-15, and 7,715/632,488 (1.2%) young people aged 16-24. The rate increased to age 11 in boys and age 10 in girls, reflecting age at diagnosis. Prevalence was 2.8% at age 10 (4.4% for boys; 1.1% for girls), and 2.9% at age 11 (4.5% for boys; 1.1% for girls). 22.0% of children and 25.5% of young people with autism reported poor health, compared with 2.0% and 4.4% without autism. Autism had OR=11.3 (11.0-11.7) in predicting poor health. Autistic females had poorer health than autistic males; OR=1.6 (1.5-1.7).

Conclusion:

Accurate information on the proportion of autistic children and their health status is essential to accurately plan appropriate prevention and intervention measures and provide resources for those who may put demand upon services designed for autistic people.

Keywords: autism, general health, children, young people, prevalence.

For peer review only - http://bmjopen.bmj.com/site/about/guidelines.xhtml

Strengths and limitations of this study:

- Large, whole country population study
- High response rate of 94%, and systematic enquiry of everyone regarding autism and their general health status
- Results are generalisable to other child and young people populations in high-income countries
- Autism and general health status were self/proxy reported by respondents rather than each person having a clinical assessment
- 6% of records were imputed

For peer review only

For peer review only - http://bmjopen.bmj.com/site/about/guidelines.xhtml

Introduction

Reports on the prevalence of autism inevitably depend upon the criteria used. The concept of autism spectrum disorders has now broadened considerably beyond original descriptions.[1,2] and clinicians also now base their diagnosis on fewer symptoms than a decade ago.[3] Additionally, there is now increased awareness about autism. Hence the reported prevalence of autism has increased. Several systematic reviews have attempted to synthesise research studies on prevalence, with overall prevalence varying, dependent upon the studies included, e.g. their age-ranges, years the studies were conducted in (and hence criteria), data-collection methods, size, and representativeness of included studies. Even when restricted to studies published since 2000, studies selected for inclusion in the reviews have shown wide ranges in reported prevalence.[4-7] Recent reviews are summarised in Table 1.

- Insert Table 1 here –

The included age-range in studies is likely to be critical in these reported rates, related to the age at which children are diagnosed. This, however, seems to be little investigated. A California, USA study demonstrated that as well as rates of diagnosis of autism increasing, this was particularly so amongst preschool children,[8] whilst a large Swedish study found that the number of autism symptoms in children diagnosed with autism had fallen in children diagnosed at age 7-12 years, but not at age 1-6 years.[3] In the National Survey of Children's Health, USA, 259 (24.6%) of children with autism were diagnosed at younger than 3 years of age, 479 (44.5%) at 3-5 years, and 383 (30.9%) at over 5 years of age.[9] A review has suggested there remains considerable variation in age at diagnosis.[10] Further current data is clearly needed.

One reason why it is important to understand prevalence of autism, is that the health profile of children and young people with autism is thought to differ from that of typically developing children and requires interventions and supports. Hence these combined factors; knowledge of prevalence and health profile of autistic children, are

For peer review only - http://bmjopen.bmj.com/site/about/guidelines.xhtml

essential for planning and delivery of services. However, in terms of general health status of children and young people with autism, there has been very little research. A study in USA reported parent-rated general health for 895 young people with autism aged 13-25 years at baseline, at five time points across 2001-2009, but did not include a general population comparison group. General health was rated as excellent, very good, good, or fair/poor.[11] Fair/poor ratings were reported for 6.6% in 2001, 6.4% in 2003, 7.6% in 2005, 6.1% in 2007, and 6.6% in 2009.[11]

To our knowledge, no other studies have investigated reported general health status of children and young people with autism, nor drawn direct comparisons with the general population. This appears to be a major gap in our knowledge.

This study aimed to investigate, on a large scale (the entire population of a country; Scotland) (1) the prevalence of autism, and age of reporting/identifying autism in childhood, and (2) the general health status of children and young people with autism compared with those without autism.

Methods

Procedures

Approval was gained from the Scottish Government for secondary analysis of Scotland's Census, 2011 data under the auspices of a collaborative research project with National Records of Scotland.

Data source

Scotland's Census, 2011 provides information on the number and characteristics of Scotland's population and households on the census day, 27 March 2011. The census is undertaken every 10 years. It includes the whole Scottish population: people living in communal establishments (such as care homes and student halls of residence) as well as people living in private households. Scotland's Census is one of the few country censuses, and indeed it may be unique, in identifying people with autism. One householder on behalf of all occupants in private households, and manager on behalf of all occupants in communal dwellings, was required to

For peer review only

For peer review only - http://bmjopen.bmj.com/site/about/guidelines.xhtml

complete the Census information. The Census form clearly stated it is a legal requirement to complete the Census, and that not completing it, or supplying false information, can result in a £1,000 fine. The Census team conducted follow up of non-responders, and provided help to respond when that was needed, hence the high completion rate of 94%.[12]

The Census team used a Census Coverage Survey, including around 40,000 households, to estimate numbers and characteristics of the missing 6%. The Coverage Survey and Census records were deterministically matched using automated and clerical matching to check for duplicates. Individuals estimated to have been missed from the Census were then imputed using a subset of characteristics from real individuals, including information on their health. The edit and imputation methodology was adapted from the Office for National Statistics rigorous and systematic guidelines, which are available here:

http://webarchive.nationalarchives.gov.uk/20160108193745/http://www.ons.gov.uk/ons/guide-method/method-quality/survey-methodology-bulletin/smb-69/index.html

Further details are available here:

http://www.scotlandscensus.gov.uk/documents/censusresults/release1b/rel1bmethodology.pdf

Full details of the methodology and other background information on Scotland's Census 2011 are available at: http://www.scotlandscensus.gov.uk/supporting-information.

Census variables

People with autism were identified from Census question 20, which asked: 'Do you have any of the following conditions which have lasted, or are expected to last, at least 12 months? Tick all that apply'. There was a choice of 10 response options, which included: Developmental disorder (for example, Autistic Spectrum Disorder or Asperger's Syndrome), Learning disability (for example, Down's Syndrome), Learning difficulty (for example, dyslexia), and Mental health condition.

For peer review only - http://bmjopen.bmj.com/site/about/guidelines.xhtml

During the methodology development for Scotland's Census, 2011, Ipsos MORI Scotland was commissioned to undertake cognitive question testing on question 20 on long-term health conditions and disabilities. This was to test whether the questions were answered accurately and willingly by respondents, and to identify any changes needed to improve data quality and/or the acceptability of the response options. Cognitive interviewing is a widely used approach to critically evaluate and improve survey questionnaires.[13] It enables researchers to modify survey material to enhance clarity. Retrospective probing was selected as the most appropriate technique. The questions were tested with 102 participants with a mix of gender, age and health conditions and disabilities (including people with more than one of the conditions), to ensure accurate and willing completion. They included people with autism, intellectual disabilities, dyslexia, dyspraxia, speech impairment, mental health conditions (both milder and more serious), and other long-term conditions. This resulted in a redesign of the question on autism, to 'Developmental disorder, (for example Autism Spectrum Disorder or Asperger's Syndrome)' in order to accurately capture specifically the data on autism. The questions on the other conditions tested (some of which, from a medical perspective, can be considered as developmental disorders) did not require any modification. Further information can be found at:

http://www.scotlandscensus.gov.uk/documents/research/2011-census-health-disability-questions.pdf

http://www.scotlandscensus.gov.uk/documents/legislation/changes-to-gov-statement-report.pdf

Hence the choice of wording of the question on autism was informed and carefully considered. The term developmental disorder was used and only prompted respondents to reply with regards to autistic spectrum disorder or Asperger syndrome, and the question distinguished autism from learning disability (which in the UK is synonymous with the international term 'intellectual disabilities'), learning difficulties such as dyslexia, and mental health conditions.

For peer review only - http://bmjopen.bmj.com/site/about/guidelines.xhtml

The Census team imputed answers for the 14.7% who did not tick any of the boxes in question 20, based on their free text answers for this question and answers to other health questions in the Census, which increased the completion rate to 97.4%. For the remaining 2.6%, the Census team assumed the most plausible explanation was that the person had no long-term condition but did not see the 'No condition' check box at the end of the question, and hence recorded them as such.

Information on general health status was collected through question 19 which had a five-point response scale: 'How is your health in general?' (1) very good, (2) good, (3) fair, (4) bad, (5) very bad. Similarly, as for question 20, question 19 was tested during the cognitive question testing during the development of the Census. The question was found to not require any modification.

Data analysis

We calculated the number and percentage of children reported to have autism, by age and gender. We also calculated the number and percentage of children and young people with and without autism reporting very good, good, fair, bad, and very bad health, and compared differences using chi-square tests. Within the whole population of children and young people in Scotland, we then used a logistic regression to calculate odds ratios (OR; with 95% confidence intervals) of autism predicting a derived, dichotomised variable of poor health (fair, bad, or very bad health) versus good health (very good or good health), adjusted for age and gender. Age was categorised into groups of 0-15 years (children), or 16-24 years (youth), with the 0-15-year olds being the reference group. The age groups were selected as in Scotland full legal capacity, with some limitations, is granted to people aged 16 and over. Gender was binary; the reference group was male. We then calculated the ORs of age and gender in predicting poor health within the population with autism. All analyses were conducted with SPSS software version 22.

Patient and Public Involvement

The question on autism was included in Scotland's Census, 2011 at the behest of third sector organisations for people with autism. People with autism took part in the cognitive question testing during the planning of the Census. This study was

For peer review only - http://bmjopen.bmj.com/site/about/guidelines.xhtml

undertaken by the Scottish Learning Disabilities Observatory, which has a specific remit for people with autism; its steering group includes partners from third sector organisations. Results from this study will be disseminated for people with autism in easy-read version via the Scottish Learning Disabilities Observatory website and newsletters.

Results

Number (%) of children and young people with autism by age and gender

Scotland's Census, 2011 includes records on 916,331 children aged 0-15 years and 632,488 young people aged 16-24 years. Autism was reported for 17,348 (1.9%) of the children, and 7,715 (1.2%) of the young people. Table 2 and Figure 1 show the age and gender distribution of the children with and without autism. As expected, there are more males than females with autism; 13,841/17,348 (79.8%) of children with autism were male. The rate of reported autism increased to age 11 in boys and age 10 in girls, being relatively similar across ages 9-15 years for both genders, reflecting the ages at which the autism was diagnosed in the population. Prevalence was 2.8% at age 10 years (4.4% for boys and 1.1% for girls), and 2.9% at age 11 years (4.5% for boys and 1.1% for girls).

- Insert Table 2 here -

- Insert Figure 1 here -

General health

Table 3 shows reported general health status of children and young people with and without autism in Scotland. The children and young people with autism reported poorer health; 22.0% of children and 25.5% of young people with autism reported poor (fair, bad, or very bad) general health, compared with only 2.0% of children and 4.4% of young people without autism (□^{2}=29365.6; df=1; p<0.001 for children, and □^{2}=7652.1; df=1; p<0.001 for young people). Table 3 shows that the discrepancy between those with and without autism was greater for females than males, for children rather than young people, and was even more prominent when comparing

For peer review only

bad/very bad health (as opposed to fair/bad/very bad health), e.g. 9.1% of girls with autism had bad/very bad health compared with only 0.4% of girls without autism.

- Insert Table 3 here -

Table 4 shows the results from the regression with the whole population data. Autism had OR=11.3 (11.0-11.7, 95% CI) in predicting poor health, adjusted for gender and age. Young people were more likely to have poor health than children, as were females. This pattern was also seen within the autistic population, more markedly so for females, and less so for increasing age when compared with the whole population (Table 5). Female gender had OR=1.6 (1.5-1.7, 95% CI), and age 16-24 years had OR=1.2 (1.1-1.3, 95% CI) in predicting poor health within the autistic population.

- Insert Table 4 here –

- Insert Table 5 here -

Discussion

Principle findings and interpretation

We identified the prevalence of reported autism to be 1.9% in children aged 0-15 years overall, and that the reported rate increased with age up to age 10 years in girls and 11 years in boys, reflecting the age at which it was identified/diagnosed. Almost all were identified by age 9 years, with the majority before primary school. Prevalence was 2.8% at age 10 years, and 2.9% at age 11 years; higher than when the rate is reported for all children overall. This is of importance when interpreting prevalence studies, as autism in early childhood will clearly be under-reported so lowering the overall reported childhood prevalence, unless detailed individual assessments are undertaken which is not realistic in large scale population-based research. Our study is the only whole-country population study we are aware of to-date to report prevalence of autism using current concepts of the autism spectrum and is highly representative as autism was systematically enquired about for the

For peer review only - http://bmjopen.bmj.com/site/about/guidelines.xhtml

entire population, with a 94% response rate. Of considerable significance, we are first to report that children/young people with autism were more than 11 times more likely to have poor health than the rest of the population. This inequality was greater for females than males, and more so than in the general population.

Comparison with existing literature

We found a higher rate of autism than that in the most recent systematic reviews on the subject. This finding most likely reflects that the data is more recent (2011) compared to the most recent reviews, which included data from studies completed a decade earlier, and that we report by year of age, rather than just for all children combined. More comparable studies include the Stockholm Youth Cohort which reported rates of autism in 2011 of 0.40% at age 0-5 years, 1.74% at age 6-12 years, 2.46% at age 13-17 years, and 1.76% at age 18-27 years; and of 1.44% at ages 0-17 years overall.[14] The Data Resource Center for Child & Adolescent Health findings for 2014[15] and 2016[9] report higher prevalence of autism at 2.2% (n=243) and 2.5% (n=1,131) in all 3-17-year olds but is on a smaller scale. The Autism and Developmental Disabilities Monitoring Network, in 11 sites in the USA, provides estimates of the prevalence of autism in 8-year-old children.[16] In 2014 this varied across sites from 1.3% to 2.9%, with a combined prevalence of 1.7%.[16]

Reported general health was substantially poorer for children and young people with autism compared with the general population. However, there is limited previous research with which to compare our findings; indeed, we believe we are the first to study general health status compared directly with the general population in a large, representative population of children and young people with autism. Our findings of poor (fair, bad, or very bad) health in 2.0% of children and 4.4% of young people without autism are similar to those reported in a National Health Interview Survey in 2014 which found fair/poor health for 1.6% (n=234) of children aged 0-17 years.[15] However, it did not report health status separately for children with autism. A further USA study reported lower rates of fair/poor health than the 25.5% we found in the young people with autism.[11] It reported fair/poor health in 6.6% in 2001, 6.4% in 2003, 7.6% in 2005, 6.1% in 2007, and 6.6% in 2009 of 895 young people with autism aged 13-25 years at baseline, but did not have a general population comparison group.[11] However, it used measures of health not directly comparable

For peer review only - http://bmjopen.bmj.com/site/about/guidelines.xhtml

with our study, using a four-point scale of excellent, very good, good, and fair/poor health.[11]

Young people with autism had poorer health than children with autism, but the extent of this difference was much less than that seen in the general population. The difference in the extent of influence of age category between the people with and without autism lies in the substantial inequalities in general health that are associated with having autism, regardless of age.

Strengths and limitations

This large-scale study covers the whole population of Scotland and we believe it is currently unique in being a whole country study in which every citizen was systematically enquired about regarding having autism and their general health status. It also had a high completion rate at 94%, suggesting the results are highly representative and likely to be generalisable to other high-income countries. The wording of the question on autism was informed in advance by the cognitive question testing procedure. It included the terms autistic spectrum disorder and Asperger's syndrome, and was distinguished from intellectual disabilities, specific learning disability, and mental health conditions. Hence, we consider that respondents will have replied accordingly, i.e. responded regarding autism. However, we have no means to check this. Respondents reported whether or not each child/young person was known to have autism rather than each person having an assessment for autism, so some reporting error is possible. The majority of reports were proxy-reports by parents, but we do not know the extent of proxy versus self-reports for the young people. Neither do we know the extent to which proxy-reporting of general health status compares with an individual's report, and the general health status responses were subjective rather than objective measurements. Whilst we described the imputation process, we cannot state with certainty whether the imputed 6% of records contained the same, more or fewer proportion of children and young people with reported autism but note that this missing 6% is a small proportion overall. Imputation of zero by the Census team on the 2.6% with missing data on long-term conditions was not tested, though considered to be the most plausible explanation. Despite these limitations, we believe the results of this study are generalisable to

For peer review only - http://bmjopen.bmj.com/site/about/guidelines.xhtml

other high-income countries and fill a significant gap in existing research on general health status of children and young people with autism.

Implications for clinicians

It is essential to have accurate information on the proportion of children and young people who are known to have autism, and their health status, in order to accurately plan appropriate prevention and intervention measures, and provision of resources for those people who may put demand upon services designed for people with autism. This requires a full understanding of age differences, and age at diagnosis. The poor general health status observed in the population of children and young people with autism demonstrates a clear need to focus on improvements in healthcare and supports, and the wider determinants of health in this group, which may well differ from the general population.

Word count: 3,024

For peer review only

Acknowledgements:

We thank the National Records of Scotland for assisting with the data analysis and dissemination stages of the project.

Contributors:

ER analysed the data, jointly interpreted it, and wrote the first draft of the manuscript, LAH-M, CG, and AH jointly interpreted the data, and contributed to the manuscript, CM and JR worked on the Census, jointly interpreted the data, and contributed to the manuscript, S-AC conceived the project, interpreted the data, and contributed to the manuscript. All authors approved the final version of the manuscript. S-AC is the study guarantor.

S-AC confirms the manuscript is an honest, accurate and transparent account of the study being reported, that no important aspects of the study have been omitted, and there has been no discrepancies from the study as planned.

Funding:

This study was funded by the Medical Research Council (grant reference MC_PC_17217) and the Scottish Government via the xxx. The funders had no role in the study design, collection, analyses and interpretation of data, in writing the report, nor in the decision to submit the article for publication.

Competing interests:

All authors have completed the Unified Competing Interest form (available on request from the corresponding author) at www.icmje.org/coi_disclosure.pdf and declare: all authors had financial support from the Scottish Government for the submitted work; no financial relationships with any organisations that might have an interest in the submitted work in the previous three years; no other relationships or activities that could appear to have influenced the submitted work.

Patient consent:

Not applicable

Ethical approval:

Permission to access data was granted by the Scottish Government.

Provenance and peer review:

For peer review only - http://bmjopen.bmj.com/site/about/guidelines.xhtml

Not commissioned; externally peer reviewed.

Data sharing statement:

Data available at:

http://www.scotlandscensus.gov.uk/ods-web/data-warehouse.html#additionaltab

For peer review only

For peer review only - http://bmjopen.bmj.com/site/about/guidelines.xhtml

References

1. Kanner L. Autistic disturbances of affective contact. *Nervous Child* 1943;2:217-250.
2. Asperger H. Die 'autistischen psychopathen' im kindersalter (autistic psychopathogy of childhood). *Archiv für Psychiatrie und Nervenkrankenheiten* 1944;177:76-136.
3. Arvidsson O, Gillberg C, Lichtenstein P, et al. Secular changes in the symptom level of clinically diagnosed autism. *J Child Psychol Psychiatry* 2018; doi:10.1111/jcpp.12864
4. French LR, Bertone A, Hyde KL, et al. Epidemiology of Autism Spectrum Disorders. In: Buxbaum J, Hof PR, eds. *The Neuroscience of Autism Spectrum Disorders*. New York: Academic Press 2013.
5. Elsabbagh M, Divan G, Koh YJ, et al. Global Prevalence of Autism and Other Pervasive Developmental Disorders. *Autism Res* 2012;5:160-179.
6. Tsai LY. Impact of DSM-5 on epidemiology of Autism Spectrum Disorder. *Res Autism Spectr Disord* 2014;8(11):1454-1470.
7. Adak R and Halder S. Systematic review on prevalence for autism spectrum disorder with respect to gender and socio-economic status. *J Ment Disord Treat* 2017;3(1):1-9.
8. Hertz-Picciotto I and Delwiche L. The Rise in Autism and the Role of Age at Diagnosis. *Epidemiol* 2009;20(1):84-90.
9. Data Resource Center for Child & Adolescent Health. *National Survey of Children's Health; 2016*. Available at: http://childhealthdata.org/browse/survey
10. Daniels AM and Mandell DS. Explaining differences in age at autism spectrum disorder diagnosis: A critical review. *Autism* 2014;18(5):583-597.
11. National Longitudinal Transition Study 2 (NLTS2). *Data Tables NLTS2 Waves 1 – 5; 2017*. Available at: http://www.nlts2.org/data_tables/index.html
12. National Records of Scotland (NRS). *2011 Census Release 1C - How the 2011 Census population estimates were obtained*. Edinburgh: National Records of Scotland 2013.
13. Wills GB. *Cognitive Interviewing. A tool for improving questionnaire design.* Thousand Oaks: Sage Publications 2005.
14. Idring S, Lundberg M, Sturn H, et al. Changes in prevalence of autism spectrum disorders in 2001–2011: Findings from the Stockholm Youth Cohort. *J Autism Dev Disord* 2015;45: 1766–1773.
15. Data Resource Center for Child & Adolescent Health. *National Health Interview Survey (NHIS) – Child; 2014.* Available at: *http://childhealthdata.org/browse/survey*
16. Centres for Disease Control and Prevention. Prevalence of Autism Spectrum Disorder Among Children Aged 8 Years - Autism and Developmental Disabilities Monitoring Network, 11 Sites, United States, 2014. *Surveill Summ* 2018;67(6):1-23.

For peer review only - http://bmjopen.bmj.com/site/about/guidelines.xhtml

Table 1. Examples of findings from systematic reviews of recent studies on childhood/youth prevalence of autism

Review		N of studies	Publication dates of studies	Median prevalence /1,000	Range /1,000
**Autistic disorder**					
French et al., 2013	Autistic disorder	26	2000-2011	2.2	0.8-9.4
	Asperger syndrome*	13	1998-2011	2.1	0.5-2.8
Elsabbagh et al., 2012	Northern European	16	2000-2008	1.9	0.7-3.9
	Western Pacific	12	2000-2011	1.2	0.3-9.4
	South East Asia/East Mediterranean	0	-	-	-
	Americas	7	2001-2010	2.2	1.1-4.1
	Overall			1.7	0.3-9.4
Tsai, 2014		43	2001-2013	2.8	0.3-19.0
**Pervasive developmental disorder**					
French et al., 2013		34	2000-2011	6.2	0.6-26.4
Elsabbagh et al., 2012	Northern Europe	14	2000-2011	6.2	3.0-11.6
	Western Pacific	4	2004-2011	-	1.6-19.0
	South East Asia/East Mediterranean	4	2007-2012	-	0.1-10.7
	Americas	13	2001-2010	6.6	1.3-11.0
	Overall			6.2	0.1-19.0
Tsai, 2014		59	2000-2014	7.0	0.2-26.4
Adak & Halder, 2017		25	2005-2015	9.2	0.7-26.4

*The authors comment on dubious quality of results

For peer review only - http://bmjopen.bmj.com/site/about/guidelines.xhtml

For peer review only

Table 2. Identified prevalence of childhood autism by age and gender

Age in years	All children			Children with autism		
	Total	Female	Male	Total	Female	Male
**0**	58,715	28,823	29,892	76 (0.1%)	34 (0.1%)	42 (0.1%)
**1**	59,556	29,188	30,368	126 (0.2%)	52 (0.2%)	74 (0.2%)
**2**	58,909	28,936	29,973	301 (0.5%)	87 (0.3%)	214 (0.7%)
**3**	58,764	28,735	30,029	509 (0.9%)	132 (0.5%)	377 (1.3%)
**4**	56,877	27,915	28,962	730 (1.3%)	176 (0.6%)	554 (1.9%)
**5**	55,224	26,910	28,314	966 (1.7%)	223 (0.8%)	743 (2.6%)
**6**	55,236	26,872	28,364	1,053 (1.9%)	200 (0.7%)	853 (3.0%)
**7**	53,786	26,172	27,614	1,154 (2.1%)	244 (0.9%)	910 (3.3%)
**8**	52,325	25,665	26,660	1,243 (2.4%)	222 (0.9%)	1,021 (3.8%)
**9**	53,046	26,022	27,024	1,418 (2.7%)	257 (1.0%)	1,161 (4.3%)
**10**	55,067	26,950	28,117	1,549 (2.8%)	306 (1.1%)	1,243 (4.4%)
**11**	56,769	27,699	29,070	1,623 (2.9%)	313 (1.1%)	1,310 (4.5%)
**12**	58,656	28,412	30,244	1,665 (2.8%)	324 (1.1%)	1,341 (4.4%)
**13**	59,971	29,353	30,618	1,705 (2.8%)	330 (1.1%)	1,375 (4.5%)
**14**	61,152	29,586	31,566	1,658 (2.7%)	307 (1.0%)	1,351 (4.3%)
**15**	62,278	29,987	32,291	1,572 (2.5%)	300 (1.0%)	1,272 (3.9%)
**0-15**	916,331	447,225	469,106	17,348 (1.9%)	3,507 (0.8%)	13,841 (3.0%)

For peer review only

For peer review only - http://bmjopen.bmj.com/site/about/guidelines.xhtml

Table 3. General health status of children and young people with and without autism

General health	Age in years											
	0-15 years N=916,331						16-24 years N=632,488					
	Autism			Without autism			Autism			Without autism		
	Total 17,348 (100%)	F 3,507 (100%)	M 13,841 (100%)	Total 898,983 (100%)	F 443,718 (100%)	M 455,265 (100%)	Total 7,715 (100%)	F 1,676 (100%)	M 6,039 (100%)	Total 624,773 (100%)	F 313,929 (100%)	M 310,844 (100%)
**Very good**	7,470 (43.1%)	1,291 (36.8%)	6,179 (44.6%)	758,328 (84.4%)	376,945 (85.0%)	381,383 (83.8%)	3,070 (39.8%)	531 (31.7%)	2,539 (42.0%)	459,492 (73.5%)	223,178 (71.1%)	236,314 (76.0%)
**Good**	6,073 (35.0%)	1,178 (33.6%)	4,895 (35.4%)	122,814 (13.7%)	58,499 (13.2%)	64,315 (14.1%)	2,683 (34.8%)	605 (36.1%)	2,078 (34.4%)	137,956 (22.1%)	75,489 (24.0%)	62,467 (20.1%)
**Fair**	2,892 (16.7%)	718 (20.5%)	2,174 (15.7%)	14,760 (1.6%)	6,800 (1.5%)	7,960 (1.7%)	1,451 (18.8%)	367 (21.9%)	1,084 (17.9%)	22,102 (3.5%)	12,507 (4.0%)	9,595 (3.1%)
**Bad**	651 (3.8%)	204 (5.8%)	447 (3.2%)	2,367 (0.3%)	1,159 (0.3%)	1,208 (0.3%)	375 (4.9%)	125 (7.5%)	250 (4.1%)	4,237 (0.7%)	2,279 (0.7%)	1,958 (0.6%)
**Very bad**	262 (1.5%)	116 (3.3%)	146 (1.1%)	714 (0.1%)	315 (0.1%)	399 (0.1%)	136 (1.8%)	48 (2.9%)	88 (1.5%)	986 (0.2%)	476 (0.2%)	510 (0.2%)

For peer review only

For peer review only - http://bmjopen.bmj.com/site/about/guidelines.xhtml

Table 4. Odds ratio of autism, age, and gender in predicting poor health* in the whole population

Variable		Odds ratio	95% confidence interval
Autism	No autism (reference)	-	
	Autism	11.339	10.983-11.707
Age	0-15 (reference)	-	
	16-24	2.137	2.098-2.176
Gender	Male (reference)	-	
	Female	1.126	1.106-1.147
Constant		.020	

*fair, bad or very bad health

Table 5. Odds ratios of age and gender in predicting poor health* in the population with autism

Variable		Odds ratio	95% confidence interval
Age	0-15 (reference)	-	
	16-24	1.206	1.133-1.284
Gender	Male (reference)	-	
	Female	1.635	1.527-1.750
Constant		.252	

*fair, bad or very bad health

For peer review only

For peer review only - http://bmjopen.bmj.com/site/about/guidelines.xhtml

Figure 1. Identified childhood prevalence of autism by age and gender

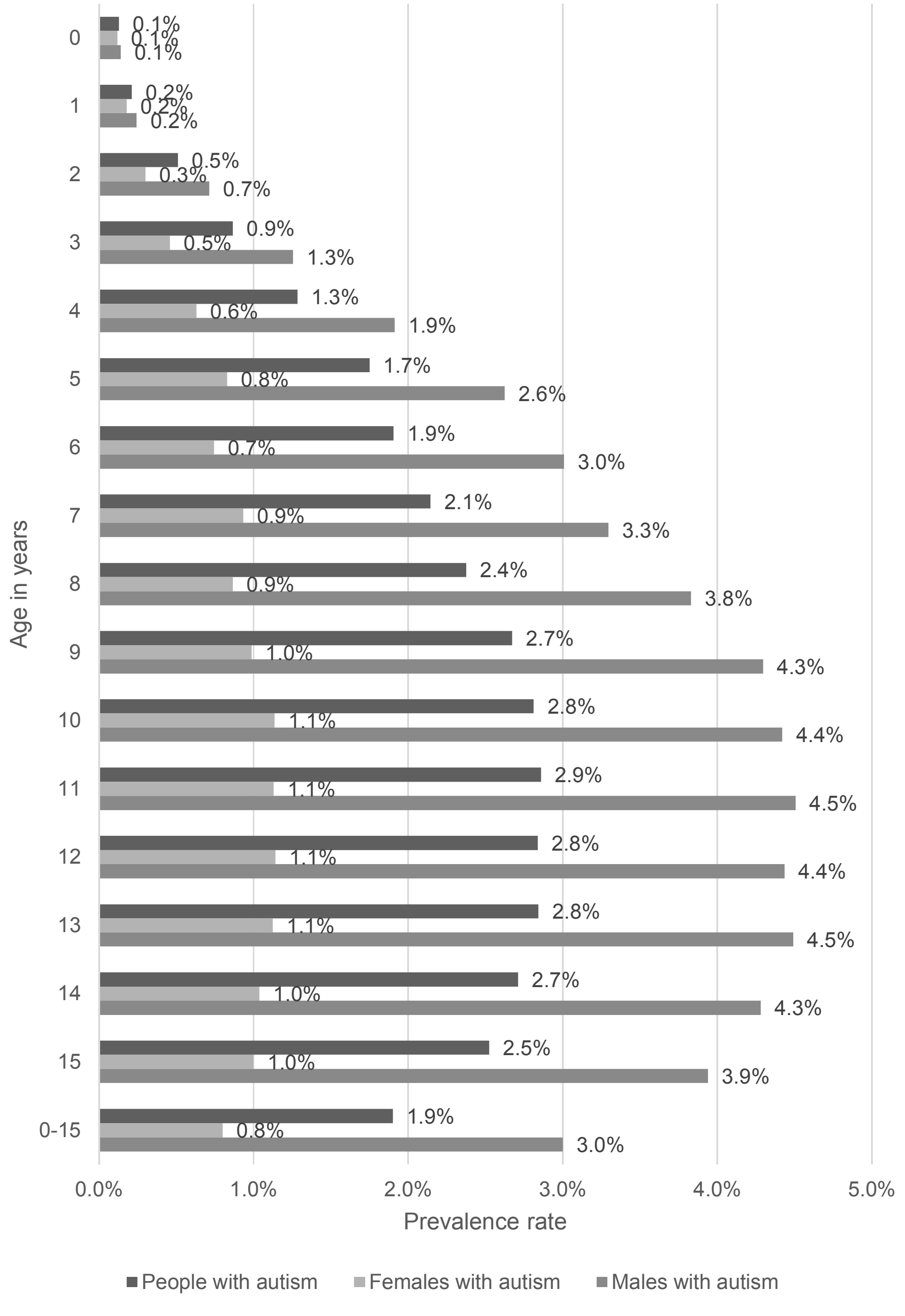

STROBE 2007 (v4) Statement—Checklist of items that should be included in reports of *cross-sectional studies*

Section/Topic	Item #	Recommendation	Reported on page #
**Title and abstract**	1	(*a*) Indicate the study's design with a commonly used term in the title or the abstract	Page 1
		(*b*) Provide in the abstract an informative and balanced summary of what was done and what was found	Page 2
**Introduction**			
Background/rationale	2	Explain the scientific background and rationale for the investigation being reported	Page 4-5 Section: Introduction
Objectives	3	State specific objectives, including any prespecified hypotheses	Page 5 Section: Introduction
**Methods**			
Study design	4	Present key elements of study design early in the paper	Page 5-8 Sections: Methods/Procedures, Data source, Census variables
Setting	5	Describe the setting, locations, and relevant dates, including periods of recruitment, exposure, follow-up, and data collection	Page 5-6 Sections: Methods/Procedures, Data source
Participants	6	(*a*) Give the eligibility criteria, and the sources and methods of selection of participants	Page 5-8 Sections: Methods/Data source, Census variables
Variables	7	Clearly define all outcomes, exposures, predictors, potential confounders, and effect modifiers. Give diagnostic criteria, if applicable	Page 6-8 Section: Methods/Census variables
Data sources/ measurement	8*	For each variable of interest, give sources of data and details of methods of assessment (measurement). Describe comparability of assessment methods if there is more than one group	Page 6-8 Section: Methods/Census variables
Bias	9	Describe any efforts to address potential sources of bias	Page 5-8

For peer review only - http://bmjopen.bmj.com/site/about/guidelines.xhtml

			Section: Methods
Study size	10	Explain how the study size was arrived at	Page 5-8<br>Sections: Methods/Data source, Census variables
Quantitative variables	11	Explain how quantitative variables were handled in the analyses. If applicable, describe which groupings were chosen and why	Page 8<br>Section: Methods/Data analysis
Statistical methods	12	(*a*) Describe all statistical methods, including those used to control for confounding	Page 8<br>Section: Methods/Data analysis
		(*b*) Describe any methods used to examine subgroups and interactions	Page 8<br>Section: Methods/Data analysis
		(*c*) Explain how missing data were addressed	Page 5-8<br>Sections: Methods/Data source, Census variables
		(*d*) If applicable, describe analytical methods taking account of sampling strategy	N/A
		(*e*) Describe any sensitivity analyses	N/A
**Results**			
Participants	13*	(a) Report numbers of individuals at each stage of study—eg numbers potentially eligible, examined for eligibility, confirmed eligible, included in the study, completing follow-up, and analysed	Page 9<br>Section: Results/Number (%) of children and young people with autism by age and gender<br>Page 17 Table 2<br>Page 20 Figure 1
		(b) Give reasons for non-participation at each stage	N/A
		(c) Consider use of a flow diagram	N/A
Descriptive data	14*	(a) Give characteristics of study participants (eg demographic, clinical, social) and information on exposures and potential confounders	Page 9<br>Section: Results/Number (%) of children and young

For peer review only

For peer review only - http://bmjopen.bmj.com/site/about/guidelines.xhtml

			people with autism by age and gender<br>Page 17 Table 2<br>Page 20 Figure 1
		(b) Indicate number of participants with missing data for each variable of interest	Page 6-8<br>Sections: Methods/ Census variables
Outcome data	15*	Report numbers of outcome events or summary measures	N/A
Main results	16	(*a*) Give unadjusted estimates and, if applicable, confounder-adjusted estimates and their precision (e.g., 95% confidence interval). Make clear which confounders were adjusted for and why they were included	Page 9-10<br>Section: Results/General health<br>Page 18 Table 3<br>Page 19 Tables 4 and 5
		(*b*) Report category boundaries when continuous variables were categorized	Page 8<br>Section: Methods/Data analysis<br>Page 17 Table 2<br>Pages 18 Table 3<br>Page 19 Tables 4 and 5<br>Page 20 Figure 1
		(*c*) If relevant, consider translating estimates of relative risk into absolute risk for a meaningful time period	N/A
Other analyses	17	Report other analyses done—eg analyses of subgroups and interactions, and sensitivity analyses	N/A
**Discussion**			
Key results	18	Summarise key results with reference to study objectives	Page 10-12<br>Section: Discussion/ Principal findings and interpretation,<br>Comparison with existing literature
Limitations	19	Discuss limitations of the study, taking into account sources of potential bias or imprecision. Discuss both direction and magnitude of any potential bias	Page 12<br>Section: Strengths and

For peer review only - http://bmjopen.bmj.com/site/about/guidelines.xhtml

			limitations
Interpretation	20	Give a cautious overall interpretation of results considering objectives, limitations, multiplicity of analyses, results from similar studies, and other relevant evidence	Page 10-12<br>Section: Discussion/ Principal findings and interpretation, Comparison with existing literature
Generalisability	21	Discuss the generalisability (external validity) of the study results	Page 12-13<br>Section: Implications for clinicians
**Other information**			
Funding	22	Give the source of funding and the role of the funders for the present study and, if applicable, for the original study on which the present article is based	Page 14<br>Section: Funding

*Give information separately for exposed and unexposed groups.

Note: An Explanation and Elaboration article discusses each checklist item and gives methodological background and published examples of transparent reporting. The STROBE checklist is best used in conjunction with this article (freely available on the Web sites of PLoS Medicine at http://www.plosmedicine.org/, Annals of Internal Medicine at http://www.annals.org/, and Epidemiology at http://www.epidem.com/). Information on the STROBE Initiative is available at www.strobe-statement.org.

For peer review only - http://bmjopen.bmj.com/site/about/guidelines.xhtml

For peer review only

BMJ Open

Age at identification, prevalence, and general health of children with autism - observational study of a whole country population

Journal:	*BMJ Open*
Manuscript ID	bmjopen-2018-025904.R1
Article Type:	Research
Date Submitted by the Author:	10-Apr-2019
Complete List of Authors:	Rydzewska, Ewelina; University of Glasgow Mental Health and Wellbeing, Institute of Health and Wellbeing<br>Hughes-McCormack, Laura; University of Glasgow Mental Health and Wellbeing, Institute of Health and Wellbeing<br>Gillberg, Christopher; University of Glasgow Institute of Health and Wellbeing<br>Henderson, Angela; University of Glasgow Mental Health and Wellbeing, Institute of Health and Wellbeing<br>MacIntyre, Cecilia; National Records of Scotland<br>Rintoul, Julie; Scottish Government Health and Social Care Analysis<br>Cooper, Sally-Ann; University of Glasgow Mental Health and Wellbeing
**Primary Subject Heading**:	Public health
Secondary Subject Heading:	Epidemiology
Keywords:	autism, general health, children, young people, prevalence

SCHOLARONE™
Manuscripts

Age at identification, prevalence, and general health of children with autism - observational study of a whole country population

Ewelina Rydzewska, Laura A Hughes-McCormack, Christopher Gillberg, Angela Henderson, Cecilia MacIntyre, Julie Rintoul, Sally-Ann Cooper

Institute of Health and Wellbeing, University of Glasgow, Mental Health and Wellbeing Group, 1st Floor, Administrative Building, Gartnavel Royal Hospital, 1055 Great Western Road, Glasgow G12 0XH, UK
Ewelina Rydzewska
Research Associate

Institute of Health and Wellbeing, University of Glasgow, Mental Health and Wellbeing Group, 1st Floor, Administrative Building, Gartnavel Royal Hospital, 1055 Great Western Road, Glasgow G12 0XH, UK
Laura A Hughes-McCormack
Research Assistant

1 Göteborgs Universitet/University of Gothenburg, Gillbergcentrum/Gillberg Neuropsychiatry Centre, Kungsgatan 12, S-411 19 Göteborg, Sweden
2 Institute of Health and Wellbeing, University of Glasgow, Mental Health and Wellbeing Group, 1st Floor, Administrative Building, Gartnavel Royal Hospital, 1055 Great Western Road, Glasgow G12 0XH, UK
Christopher Gillberg
Professor of Child and Adolescent Psychiatry

Institute of Health and Wellbeing, University of Glasgow, Mental Health and Wellbeing Group, 1st Floor, Administrative Building, Gartnavel Royal Hospital, 1055 Great Western Road, Glasgow G12 0XH, UK
Angela Henderson
Scottish Learning Disabilities Observatory Deputy Director

Census User Needs, Content and Benefits, National Records of Scotland, Ladywell House, Edinburgh EH12 7TF, UK
Cecilia MacIntyre
Statistician

Health & Social Care Analysis, Scottish Government, St. Andrew's House, Regent Road, Edinburgh EH1 3DG, UK
Julie Rintoul
Statistician

Institute of Health and Wellbeing, University of Glasgow, Mental Health and Wellbeing Group, 1st Floor, Administrative Building, Gartnavel Royal Hospital, 1055 Great Western Road, Glasgow G12 0XH, UK
Sally-Ann Cooper
Professor of Learning Disabilities

Corresponding author:
Professor Sally-Ann Cooper
Email: Sally-Ann.Cooper@glasgow.ac.uk

For peer review only - http://bmjopen.bmj.com/site/about/guidelines.xhtml

Abstract

Objectives:

Reported childhood prevalence of autism varies considerably between studies and over time, and general health status has been little investigated. We aimed to investigate contemporary prevalence of reported autism by age, and general health status of children/young people with and without autism.

Design:

Secondary analysis of Scotland's Census, 2011 data. Cross-sectional study.

Setting:

General population of Scotland.

Participants:

All children (n=916,331) and young people (n=632,488) in Scotland.

Main outcome measures:

Number (%) of children/young people reported to have autism; and their general health status. Prevalence of autism; prevalence of poor health (fair, bad and very bad health); odds ratios (OR: 95% confidence intervals) of autism predicting poor health, adjusted for age and gender; and OR for age and gender in predicting poor health within the population with reported autism.

Results:

Autism was reported for 17,348/916,331 (1.9%) children aged 0-15, and 7,715/632,488 (1.2%) young people aged 16-24. The rate increased to age 11 in boys and age 10 in girls, reflecting age at diagnosis. Prevalence was 2.8% at age 10 (4.4% for boys; 1.1% for girls), and 2.9% at age 11 (4.5% for boys; 1.1% for girls). 22.0% of children and 25.5% of young people with autism reported poor health, compared with 2.0% and 4.4% without autism. Autism had OR=11.3 (11.0-11.7) in predicting poor health. Autistic females had poorer health than autistic males; OR=1.6 (1.5-1.7).

Conclusion:

Accurate information on the proportion of autistic children and their health status is essential to accurately plan appropriate prevention and intervention measures and provide resources for those who may put demand upon services designed for autistic people.

Keywords: autism, general health, children, young people, prevalence.

For peer review only

For peer review only - http://bmjopen.bmj.com/site/about/guidelines.xhtml

Strengths and limitations of this study:

- Large, whole country population study
- High response rate of 94%, and systematic enquiry of everyone regarding autism and their general health status
- Results are generalisable to other child and young people populations in high-income countries
- Autism and general health status were self/proxy reported by respondents rather than each person having a clinical assessment
- 6% of records were imputed

For peer review only

For peer review only - http://bmjopen.bmj.com/site/about/guidelines.xhtml

Introduction

Reports on the prevalence of autism inevitably depend upon the criteria used. The concept of autism spectrum disorders has now broadened considerably beyond original descriptions.[1,2] and clinicians also now base their diagnosis on fewer symptoms than a decade ago.[3] Additionally, there is now increased awareness about autism. Hence the reported prevalence of autism has increased. Several systematic reviews have attempted to synthesise research studies on prevalence, with overall prevalence varying, dependent upon the studies included, e.g. their age-ranges, years the studies were conducted in (and hence criteria), data-collection methods, size, and representativeness of included studies. Even when restricted to studies published since 2000, studies selected for inclusion in the reviews have shown wide ranges in reported prevalence.[4-7] Recent reviews are summarised in Table 1.

- Insert Table 1 here –

The included age-range in studies is likely to be critical in these reported rates, related to the age at which children are diagnosed. This, however, seems to be little investigated. A California, USA study demonstrated that as well as rates of diagnosis of autism increasing, this was particularly so amongst preschool children,[8] whilst a large Swedish study found that the number of autism symptoms in children diagnosed with autism had fallen in children diagnosed at age 7-12 years, but not at age 1-6 years.[3] In the National Survey of Children's Health, USA, 259 (24.6%) of children with autism were diagnosed at younger than 3 years of age, 479 (44.5%) at 3-5 years, and 383 (30.9%) at over 5 years of age.[9] A review has suggested there remains considerable variation in age at diagnosis.[10] Further current data is clearly needed.

One reason why it is important to understand prevalence of autism, is that the health profile of children and young people with autism is thought to differ from that of typically developing children and requires interventions and supports.[11-13] Therefore, these combined factors, i.e. knowledge of prevalence and health profile of autistic children, are essential for planning and delivery of services.

For peer review only - http://bmjopen.bmj.com/site/about/guidelines.xhtml

Subjective general health status is commonly measured in general population studies, and has been demonstrated to be extremely valid, with a strongly predictive linear gradient across health status (from best to poorest) being associated with subsequent number of medical appointments, hospital admissions, and mortality.[14-17] It is, therefore, important to measure if there are general health status differences in children and young people with autism compared with other children. However, in terms of general health status of children and young people with autism, there has been very little research. A study in USA reported parent-rated general health for 895 young people with autism aged 13-25 years at baseline, at five time points across 2001-2009, but did not include a general population comparison group.[18] General health was rated as excellent, very good, good, or fair/poor. Fair/poor ratings were reported for 6.6% in 2001, 6.4% in 2003, 7.6% in 2005, 6.1% in 2007, and 6.6% in 2009.[18] A large study presenting data from the 2011-2012 National Survey of Children's Health identified 1,188/56,746 children with autism under the age of 18, who were found to have significantly lower log odds of health (-1.30, $p<0.001$) compared to all other children.[19]

To our knowledge, no other studies have investigated reported general health status of children and young people with autism, nor drawn direct comparisons with the general population. This appears to be a major gap in our knowledge.

This study aimed to investigate, on a large scale (the entire population of a country; Scotland) (1) the prevalence of autism, and age of reporting/identifying autism in childhood, and (2) the general health status of children and young people with autism compared with those without autism.

Methods

Procedures

Approval was gained from the Scottish Government for secondary analysis of Scotland's Census, 2011 data under the auspices of a collaborative research project with National Records of Scotland.

For peer review only - http://bmjopen.bmj.com/site/about/guidelines.xhtml

Data source

Scotland's Census, 2011 provides information on the number and characteristics of Scotland's population and households on the census day, 27 March 2011. The census is undertaken every 10 years. It includes the whole Scottish population: people living in communal establishments (such as care homes and student halls of residence) as well as people living in private households. Scotland's Census is one of the few country censuses, and indeed it may be unique, in identifying people with autism. One householder on behalf of all occupants in private households, and manager on behalf of all occupants in communal dwellings, was required to complete the Census information. In the great majority of cases this was, therefore, a parent of the child/young person. The Census form clearly stated it is a legal requirement to complete the Census, and that not completing it, or supplying false information, can result in a £1,000 fine. The Census team conducted follow up of non-responders, and provided help to respond when that was needed, hence the high completion rate of 94%.[20] For 2011, the UK Census Offices endorsed CANCEIS (Canadian Census Edit and Imputation System) as the cornerstone of the 2011 Census Editing Strategy. CANCEIS performs robust, cost effective, editing and imputation whilst incorporating methodological best practice. The Census team used a Census Coverage Survey, including around 40,000 households, to estimate numbers and characteristics of the missing 6%. The Coverage Survey and Census records were deterministically matched using automated and clerical matching to check for duplicates. Individuals estimated to have been missed from the Census were then imputed using a subset of characteristics from real individuals, including information on their health. The edit and imputation methodology was adapted from the rigorous and systematic guidelines of the UK's largest independent producer of official statistics and the recognised national statistical institute of the UK.[21] Two further Scottish Government reports provide information on the estimation and adjustment process used to produce census population estimates for Scotland[22] as well as full details of the methods and other background information.[23]

Census variables

People with autism were identified from Census question 20, which asked: 'Do you have any of the following conditions which have lasted, or are expected to last, at least

For peer review only - http://bmjopen.bmj.com/site/about/guidelines.xhtml

12 months? Tick all that apply'. There was a choice of 10 response options, which included: Developmental disorder (for example, Autistic Spectrum Disorder or Asperger's Syndrome), Learning disability (for example, Down's Syndrome), Learning difficulty (for example, dyslexia), and Mental health condition.

During the methodology development for Scotland's Census, 2011, Ipsos MORI Scotland was commissioned to undertake cognitive question testing on question 20 on long-term health conditions and disabilities. This was to test whether the questions were answered accurately and willingly by respondents, and to identify any changes needed to improve data quality and/or the acceptability of the response options for the Scottish population. Cognitive interviewing is a widely used approach to critically evaluate and improve survey questionnaires.[24] It enables researchers to modify survey material to enhance clarity. Retrospective probing was selected as the most appropriate technique. The questions were tested with 102 participants with a mix of gender, age and health conditions and disabilities (including people with more than one of the conditions), to ensure accurate and willing completion.[25] They included people with autism, intellectual disabilities, dyslexia, dyspraxia, speech impairment, mental health conditions (both milder and more serious), and other long-term conditions. This resulted in a redesign of the question on autism, to 'Developmental disorder, (for example Autism Spectrum Disorder or Asperger's Syndrome)' in order to accurately capture specifically the data on autism. The questions on the other conditions tested (some of which, from a medical perspective, can be considered as developmental disorders) did not require any modification.
Hence the choice of wording of the question on autism was informed and carefully considered. The term developmental disorder was used and only prompted respondents to reply with regards to autistic spectrum disorder or Asperger syndrome, and the question distinguished autism from learning disability (which in the UK is synonymous with the international term 'intellectual disabilities'), learning difficulties such as dyslexia, and mental health conditions.

The Census team imputed answers for the 14.7% who did not tick any of the boxes in question 20, based on their free text answers for this question and answers to other health questions in the Census, which increased the completion rate to 97.4%. For the

For peer review only - http://bmjopen.bmj.com/site/about/guidelines.xhtml

remaining 2.6%, the Census team assumed the most plausible explanation was that the person had no long-term condition but did not see the ‘No condition’ check box at the end of the question, and hence recorded them as such.[26]

Information on general health status was collected through question 19 which had a five-point response scale: ‘How is your health in general?’ (1) very good, (2) good, (3) fair, (4) bad, (5) very bad. Similarly, as for question 20, question 19 was tested during the cognitive question testing during the development of the Census. The question was found to not require any modification.

Data analysis

We calculated the number and percentage of children reported to have autism, by age and gender. We also calculated the number and percentage of children and young people with and without autism reporting very good, good, fair, bad, and very bad health, and compared differences using chi-square tests. Within the whole population of children and young people in Scotland, we then used a logistic regression to calculate odds ratios (OR; with 95% confidence intervals) of autism predicting a derived, dichotomised variable of poor health (fair, bad, or very bad health) versus good health (very good or good health), adjusted for age and gender. Age was categorised into groups of 0-15 years (children), or 16-24 years (youth), with the 0-15-year olds being the reference group. The age groups were selected as in Scotland full legal capacity, with some limitations, is granted to people aged 16 and over. Gender was binary; the reference group was male. We then calculated the ORs of age and gender in predicting poor health within the population with autism. All analyses were conducted with SPSS software version 22.

Patient and Public Involvement

The question on autism was included in Scotland’s Census, 2011 at the behest of third sector organisations for people with autism. People with autism took part in the cognitive question testing during the planning of the Census. This study was undertaken by the Scottish Learning Disabilities Observatory, which has a specific remit for people with autism; its steering group includes partners from third sector organisations. Results from this study will be disseminated for people with autism in

For peer review only - http://bmjopen.bmj.com/site/about/guidelines.xhtml

easy-read version via the Scottish Learning Disabilities Observatory website and newsletters.

Results

Number (%) of children and young people with autism by age and gender

Scotland's Census, 2011 includes records on 916,331 children aged 0-15 years and 632,488 young people aged 16-24 years. Autism was reported for 17,348 (1.9%) of the children, and 7,715 (1.2%) of the young people. Table 2 and Figure 1 show the age and gender distribution of the children with and without autism. As expected, there are more males than females with autism; 13,841/17,348 (79.8%) of children with autism were male. The rate of reported autism increased to age 11 in boys and age 10 in girls, being relatively similar across ages 9-15 years for both genders, reflecting the ages at which the autism was diagnosed in the population. Prevalence was 2.8% at age 10 years (4.4% for boys and 1.1% for girls), and 2.9% at age 11 years (4.5% for boys and 1.1% for girls).

- Insert Table 2 here -

- Insert Figure 1 here -

General health

Table 3 shows reported general health status of children and young people with and without autism in Scotland. The children and young people with autism reported poorer health; 22.0% of children and 25.5% of young people with autism reported poor (fair, bad, or very bad) general health, compared with only 2.0% of children and 4.4% of young people without autism ($\square^2$=29365.6; df=1; p<0.001 for children, and $\square^2$=7652.1; df=1; p<0.001 for young people). Table 3 shows that the discrepancy between those with and without autism was greater for females than males, for children rather than young people, and was even more prominent when comparing bad/very bad health (as opposed to fair/bad/very bad health), e.g. 9.1% of girls with autism had bad/very bad health compared with only 0.4% of girls without autism.

For peer review only

For peer review only - http://bmjopen.bmj.com/site/about/guidelines.xhtml

- Insert Table 3 here -

Table 4 shows the results from the regression with the whole population data. Autism had OR=11.3 (11.0-11.7, 95% CI) in predicting poor health, adjusted for gender and age. Young people were more likely to have poor health than children, as were females. This pattern was also seen within the autistic population, more markedly so for females, and less so for increasing age when compared with the whole population (Table 5). Female gender had OR=1.6 (1.5-1.7, 95% CI), and age 16-24 years had OR=1.2 (1.1-1.3, 95% CI) in predicting poor health within the autistic population.

- Insert Table 4 here –

- Insert Table 5 here -

Discussion

Principle findings and interpretation

We identified the prevalence of reported autism to be 1.9% in children aged 0-15 years overall, and that the reported rate increased with age up to age 10 years in girls and 11 years in boys, reflecting the age at which it was identified/diagnosed. Almost all were identified by age 9 years, with the majority before primary school. Prevalence was 2.8% at age 10 years, and 2.9% at age 11 years; higher than when the rate is reported for all children overall. This is of importance when interpreting prevalence studies, as autism in early childhood will clearly be under-reported so lowering the overall reported childhood prevalence, unless detailed individual assessments are undertaken which is not realistic in large scale population-based research. Our study is the only whole-country population study we are aware of to-date to report prevalence of autism using current concepts of the autism spectrum and is highly representative as autism was systematically enquired about for the entire population, with a 94% response rate. Of considerable significance, we report that children/young people with autism were more than 11 times more likely to have poor health than the rest of the population. This inequality was greater for females than males, and more so than in the general population.

For peer review only - http://bmjopen.bmj.com/site/about/guidelines.xhtml

Comparison with existing literature

We found a higher rate of autism than that in the most recent systematic reviews on the subject. This finding most likely reflects that the data is more recent (2011) compared to the most recent reviews, which included data from studies completed a decade earlier, and that we report by year of age, rather than just for all children combined. More comparable studies include the Stockholm Youth Cohort which reported rates of autism in 2011 of 0.40% at age 0-5 years, 1.74% at age 6-12 years, 2.46% at age 13-17 years, and 1.76% at age 18-27 years; and of 1.44% at ages 0-17 years overall.[27] The Data Resource Center for Child & Adolescent Health findings for 2014[28] and 2016[9] report higher prevalence of autism at 2.2% (n=243) and 2.5% (n=1,131) in all 3-17-year olds but is on a smaller scale. The Autism and Developmental Disabilities Monitoring Network, in 11 sites in the USA, provides estimates of the prevalence of autism in 8-year-old children.[29] In 2014 this varied across sites from 1.3% to 2.9%, with a combined prevalence of 1.7%.[29]

Reported general health was substantially poorer for children and young people with autism compared with the general population. However, there is limited previous research with which to compare our findings; indeed, we believe we are the first to study general health status compared directly with the general population in a whole country population of children and young people with autism. Our findings of poor (fair, bad, or very bad) health in 2.0% of children and 4.4% of young people without autism are similar to those reported in a National Health Interview Survey in 2014 which found fair/poor health for 1.6% (n=234) of children aged 0-17 years.[28] However, it did not report health status separately for children with autism. A further USA study reported lower rates of fair/poor health than the 25.5% we found in the young people with autism.[18] It reported fair/poor health in 6.6% in 2001, 6.4% in 2003, 7.6% in 2005, 6.1% in 2007, and 6.6% in 2009 of 895 young people with autism aged 13-25 years at baseline, but did not have a general population comparison group.[18] However, it used measures of health not directly comparable with our study, using a four-point scale of excellent, very good, good, and fair/poor health.[18] Our findings of odds ratio of 11.3 for autism predicting poor general health in the whole population of children and young people are not directly comparable with the findings from the National Survey of

For peer review only - http://bmjopen.bmj.com/site/about/guidelines.xhtml

Children's Health from 2011-2012, due to differences in the scales used, though the results are in the same direction.[19]

Young people with autism had poorer health than children with autism, but the extent of this difference was much less than that seen in the general population. The difference in the extent of influence of age category between the people with and without autism lies in the substantial inequalities in general health that are associated with having autism, regardless of age. Our findings show that children and young people with autism of all ages are more likely to experience poorer general health compared to the rest of the population. We are unable to explain the reasons for this, but note that it is in addition to, and may be related to, their increase in comorbidities compared with other children and young people.[11-13] This requires further investigation.

Strengths and limitations

This large-scale study covers the whole population of Scotland and we believe it is currently unique in being a whole country study in which every citizen was systematically enquired about regarding having autism and their general health status. It also had a high completion rate at 94%, suggesting the results are highly representative and likely to be generalisable to other high-income countries. Limitations include the use of the term of 'developmental disorders' in the Census. However, it prompted responses only for the examples of autistic spectrum disorder or Asperger's syndrome, and was tested prior to its use at the Census. Furthermore, the developmental disorders category was distinguished from intellectual disabilities, learning difficulties, and mental health conditions, which are important distinctions. The wording of the question on autism was informed in advance by the cognitive question testing procedure. Hence, we consider that respondents will have replied accordingly, i.e. regarding autism. However, we have no means to check this. Respondents reported whether or not each child/young person was known to have autism rather than each person having an assessment for autism. We are unable to report on the age that each child/young person received their diagnosis; hence we report instead the number of children at each age who have received the diagnosis. They are the proportion at each age who will call upon services for children/young persons with

For peer review only - http://bmjopen.bmj.com/site/about/guidelines.xhtml

autism, so this information is important for service planning. Some reporting error is possible, but we are unable to check this. The majority of reports were proxy-reports by parents, but we do not know the extent of proxy versus self-reports for the young people. Neither do we know the extent to which proxy-reporting of general health status compares with an individual's report. The general health status responses were subjective measurements, which might have been influenced by a variety of factors such as carer burden. It is controversial as to whether autism can be diagnosed in very young children. We found that a small number did report it in the first two years. Whilst there may be some reporting error, differences in development in autistic children have been reported to be apparent from as early as 6 months, and widespread by 18 months.[30] The data from this study were collected in 2011, so it will not have captured any changes that have occurred since then. Whilst we described the imputation process, we cannot state with certainty whether the imputed 6% of records contained the same, more or fewer proportion of children and young people with reported autism but note that this missing 6% is a small proportion overall. Imputation of zero by the Census team on the 2.6% of the population with missing data on long-term conditions was not tested, though considered to be the most plausible explanation. Despite these limitations, we believe the results of this study are generalisable to other high-income countries and fill a significant gap in existing research on general health status of children and young people with autism.

Implications for clinicians

It is essential to have accurate information on the proportion of children and young people who are known to have autism, and their health status, in order to accurately plan appropriate prevention and intervention measures, and provision of resources for those people who may put demand upon services designed for people with autism. This requires a full understanding of age differences, and age at diagnosis. The poor general health status observed in the population of children and young people with autism demonstrates a clear need to focus on improvements in healthcare and supports, and the wider determinants of health in this group, which may well differ from the general population.

Word count: 3,539

For peer review only

Acknowledgements:

We thank the National Records of Scotland for assisting with the data analysis and dissemination stages of the project.

Contributors:

ER analysed the data, jointly interpreted it, and wrote the first draft of the manuscript, LAH-M, CG, and AH jointly interpreted the data, and contributed to the manuscript, CM and JR worked on the Census, jointly interpreted the data, and contributed to the manuscript, S-AC conceived the project, interpreted the data, and contributed to the manuscript. All authors approved the final version of the manuscript. S-AC is the study guarantor.

S-AC confirms the manuscript is an honest, accurate and transparent account of the study being reported, that no important aspects of the study have been omitted, and there has been no discrepancies from the study as planned.

Funding:

This study was funded by the Medical Research Council (grant reference MC_PC_17217) and the Scottish Government via the xxx. The funders had no role in the study design, collection, analyses and interpretation of data, in writing the report, nor in the decision to submit the article for publication.

Competing interests:

All authors have completed the Unified Competing Interest form (available on request from the corresponding author) at www.icmje.org/coi_disclosure.pdf and declare: all authors had financial support from the Scottish Government for the submitted work; no financial relationships with any organisations that might have an interest in the submitted work in the previous three years; no other relationships or activities that could appear to have influenced the submitted work.

Patient consent:

Not applicable

Ethical approval:

Permission to access data was granted by the Scottish Government.

Provenance and peer review:

Not commissioned; externally peer reviewed.

Data sharing statement:

Data available at:

http://www.scotlandscensus.gov.uk/ods-web/data-warehouse.html#additionaltab

For peer review only - http://bmjopen.bmj.com/site/about/guidelines.xhtml

References

1. Kanner L. Autistic disturbances of affective contact. *Nervous Child* 1943;2:217-250.
2. Asperger H. Die 'autistischen psychopathen' im kindersalter (autistic psychopathogy of childhood). *Archiv für Psychiatrie und Nervenkrankenheiten* 1944;177:76-136.
3. Arvidsson O, Gillberg C, Lichtenstein P, et al. Secular changes in the symptom level of clinically diagnosed autism. *J Child Psychol Psychiatry* 2018; doi:10.1111/jcpp.12864
4. French LR, Bertone A, Hyde KL, et al. Epidemiology of Autism Spectrum Disorders. In: Buxbaum J, Hof PR, eds. *The Neuroscience of Autism Spectrum Disorders*. New York: Academic Press 2013.
5. Elsabbagh M, Divan G, Koh YJ, et al. Global Prevalence of Autism and Other Pervasive Developmental Disorders. *Autism Res* 2012;5:160-179.
6. Tsai LY. Impact of DSM-5 on epidemiology of Autism Spectrum Disorder. *Res Autism Spectr Disord* 2014;8(11):1454-1470.
7. Adak R and Halder S. Systematic review on prevalence for autism spectrum disorder with respect to gender and socio-economic status. *J Ment Disord Treat* 2017;3(1):1-9.
8. Hertz-Picciotto I and Delwiche L. The Rise in Autism and the Role of Age at Diagnosis. *Epidemiol* 2009;20(1):84-90.
9. Data Resource Center for Child & Adolescent Health. *National Survey of Children's Health; 2016*. Available at: http://childhealthdata.org/browse/survey
10. Daniels AM and Mandell DS. Explaining differences in age at autism spectrum disorder diagnosis: A critical review. *Autism* 2014;18(5):583-597.
11. Fombonne E. Epidemiological Surveys of Autism and Other Pervasive Developmental Disorders: An Update. *J Autism Dev Disord* 2003;33(4):365-382.
12. Rydzewska E, Hughes-McCormack LA, Gillberg C, et al. Prevalence of sensory impairments, physical and intellectual disabilities, and mental health in children and young people with self/proxy-reported autism: Observational study of a whole country population. *Autism* 2018; 136236131879127.
13. Simonoff E, Pickles A, Charman T, et al. Psychiatric disorders in children with autism spectrum disorders: Prevalence, comorbidity and associated factors in a population-derived sample. *J Am Acad Child Adolesc Psychiatry* 2008;47:921–929.
14. Burnstrom B and Fredlund P. Self rated health: is it as good a predictor of subsequent mortality among adults in lower as well as in higher social classes? J Epidemiol Community Health 2001;55(11):836-840
15. Heistaro S, Jousilahti P, Lahelma E, et al. Self rated health and mortality: a long term prospective study in Eastern Finland. J Epidemiol Community Health 2001;55:227-232
16. Miilunpalo S, Vuori I, Oja P, et al. Self-rated health status as a health measure: the predictive value of self-reported health status on the use of physician services

For peer review only - http://bmjopen.bmj.com/site/about/guidelines.xhtml

and on mortality in the working-age population. *J Clin Epidemiol* 1997;50(5):517-28.
17. Schnittker J and Bacak V. The increasing predictive validity of self-rated health. *PLOS One* 2014; https://doi.org/10.1371/journal.pone.0084933
18. National Longitudinal Transition Study 2 (NLTS2). *Data Tables NLTS2 Waves 1 – 5; 2017*. Available at: http://www.nlts2.org/data_tables/index.html
19. Rigles, B. The relationship between adverse childhood events, resiliency and health among children with autism. *J Autism Dev Disord* 2017;47(1):187-202.
20. National Records of Scotland (NRS). *2011 Census Release 1C - How the 2011 Census population estimates were obtained*. Edinburgh: National Records of Scotland 2013.
21. Office for National Statistics. Survey Methodology Bulletin No. 69 – September 2011. *Office for National Statistics* 2011; https://webarchive.nationalarchives.gov.uk/20160108193745/http://www.ons.gov.uk/ons/guide-method/method-quality/survey-methodology-bulletin/smb-69/index.html
22. National Records of Scotland. 2011 Census. Release 1B - How the 2011 Census population estimates were obtained. *National Records of Scotland* 2013; http://www.scotlandscensus.gov.uk/documents/censusresults/release1b/rel1bmethodology.pdf
23. National Records of Scotland. Scotland's Census 2011. Supporting information. *National Records of Scotland* 2011; http://www.scotlandscensus.gov.uk/supporting-information.
24. Wills GB. *Cognitive Interviewing. A tool for improving questionnaire design.* Thousand Oaks: Sage Publications 2005.
25. General Register Office for Scotland. 2011 Census Question Testing - The Health and Disability Questions (2009). *General Register Office for Scotland* 2009; https://www.scotlandscensus.gov.uk/documents/research/2011-census-health-disability-questions.pdf
26. National Records of Scotland. Scotland's Census 2011 Data quality issues for the long-term health conditions question. *National Records of Scotland* 2017; https://www.scotlandscensus.gov.uk/documents/supporting_information/Long_Term_Condition_Quality_Report.pdf
27. Idring S, Lundberg M, Sturn H, et al. Changes in prevalence of autism spectrum disorders in 2001–2011: Findings from the Stockholm Youth Cohort. *J Autism Dev Disord* 2015;45:1766–1773.
28. Data Resource Center for Child & Adolescent Health. *National Health Interview Survey (NHIS) – Child; 2014.* Available at: *http://childhealthdata.org/browse/survey*
29. Centres for Disease Control and Prevention. Prevalence of Autism Spectrum Disorder Among Children Aged 8 Years - Autism and Developmental Disabilities Monitoring Network, 11 Sites, United States, 2014. *Surveill Summ* 2018;67(6):1-23.
30. National Institute for Health and Clinical Excellence. Autism diagnosis in children and young people. Evidence update April 2013. A summary of selected new evidence relevant to NICE clinical guideline 128 'Autism - recognition, referral and diagnosis of children and young people on the autism spectrum' (2011). Evidence update 40. *National Institute for Health and Clinical Excellence* 2013.

For peer review only - http://bmjopen.bmj.com/site/about/guidelines.xhtml

Table 1. Examples of findings from systematic reviews of recent studies on childhood/youth prevalence of autism

Review		N of studies	Publication dates of studies	Median prevalence /1,000	Range /1,000
**Autistic disorder**					
French et al., 2013	Autistic disorder	26	2000-2011	2.2	0.8-9.4
	Asperger syndrome*	13	1998-2011	2.1	0.5-2.8
Elsabbagh et al., 2012	Northern European	16	2000-2008	1.9	0.7-3.9
	Western Pacific	12	2000-2011	1.2	0.3-9.4
	South East Asia/East Mediterranean	0	-	-	-
	Americas	7	2001-2010	2.2	1.1-4.1
	Overall			1.7	0.3-9.4
Tsai, 2014		43	2001-2013	2.8	0.3-19.0
**Pervasive developmental disorder**					
French et al., 2013		34	2000-2011	6.2	0.6-26.4
Elsabbagh et al., 2012	Northern Europe	14	2000-2011	6.2	3.0-11.6
	Western Pacific	4	2004-2011	-	1.6-19.0
	South East Asia/East Mediterranean	4	2007-2012	-	0.1-10.7
	Americas	13	2001-2010	6.6	1.3-11.0
	Overall			6.2	0.1-19.0
Tsai, 2014		59	2000-2014	7.0	0.2-26.4
Adak & Halder, 2017		25	2005-2015	9.2	0.7-26.4

*The authors comment on dubious quality of results

For peer review only - http://bmjopen.bmj.com/site/about/guidelines.xhtml

Table 2. Identified prevalence of childhood autism by age and gender

Age in years	All children			Children with autism		
	Total	Female	Male	Total	Female	Male
0	58,715	28,823	29,892	76 (0.1%)	34 (0.1%)	42 (0.1%)
1	59,556	29,188	30,368	126 (0.2%)	52 (0.2%)	74 (0.2%)
2	58,909	28,936	29,973	301 (0.5%)	87 (0.3%)	214 (0.7%)
3	58,764	28,735	30,029	509 (0.9%)	132 (0.5%)	377 (1.3%)
4	56,877	27,915	28,962	730 (1.3%)	176 (0.6%)	554 (1.9%)
5	55,224	26,910	28,314	966 (1.7%)	223 (0.8%)	743 (2.6%)
6	55,236	26,872	28,364	1,053 (1.9%)	200 (0.7%)	853 (3.0%)
7	53,786	26,172	27,614	1,154 (2.1%)	244 (0.9%)	910 (3.3%)
8	52,325	25,665	26,660	1,243 (2.4%)	222 (0.9%)	1,021 (3.8%)
9	53,046	26,022	27,024	1,418 (2.7%)	257 (1.0%)	1,161 (4.3%)
10	55,067	26,950	28,117	1,549 (2.8%)	306 (1.1%)	1,243 (4.4%)
11	56,769	27,699	29,070	1,623 (2.9%)	313 (1.1%)	1,310 (4.5%)
12	58,656	28,412	30,244	1,665 (2.8%)	324 (1.1%)	1,341 (4.4%)
13	59,971	29,353	30,618	1,705 (2.8%)	330 (1.1%)	1,375 (4.5%)
14	61,152	29,586	31,566	1,658 (2.7%)	307 (1.0%)	1,351 (4.3%)
15	62,278	29,987	32,291	1,572 (2.5%)	300 (1.0%)	1,272 (3.9%)
0-15	916,331	447,225	469,106	17,348 (1.9%)	3,507 (0.8%)	13,841 (3.0%)

For peer review only - http://bmjopen.bmj.com/site/about/guidelines.xhtml

Table 3. General health status of children and young people with and without autism

General health	Age in years											
	0-15 years N=916,331						16-24 years N=632,488					
	Autism			Without autism			Autism			Without autism		
	Total 17,348 (100%)	F 3,507 (100%)	M 13,841 (100%)	Total 898,983 (100%)	F 443,718 (100%)	M 455,265 (100%)	Total 7,715 (100%)	F 1,676 (100%)	M 6,039 (100%)	Total 624,773 (100%)	F 313,929 (100%)	M 310,844 (100%)
**Very good**	7,470 (43.1%)	1,291 (36.8%)	6,179 (44.6%)	758,328 (84.4%)	376,945 (85.0%)	381,383 (83.8%)	3,070 (39.8%)	531 (31.7%)	2,539 (42.0%)	459,492 (73.5%)	223,178 (71.1%)	236,314 (76.0%)
**Good**	6,073 (35.0%)	1,178 (33.6%)	4,895 (35.4%)	122,814 (13.7%)	58,499 (13.2%)	64,315 (14.1%)	2,683 (34.8%)	605 (36.1%)	2,078 (34.4%)	137,956 (22.1%)	75,489 (24.0%)	62,467 (20.1%)
**Fair**	2,892 (16.7%)	718 (20.5%)	2,174 (15.7%)	14,760 (1.6%)	6,800 (1.5%)	7,960 (1.7%)	1,451 (18.8%)	367 (21.9%)	1,084 (17.9%)	22,102 (3.5%)	12,507 (4.0%)	9,595 (3.1%)
**Bad**	651 (3.8%)	204 (5.8%)	447 (3.2%)	2,367 (0.3%)	1,159 (0.3%)	1,208 (0.3%)	375 (4.9%)	125 (7.5%)	250 (4.1%)	4,237 (0.7%)	2,279 (0.7%)	1,958 (0.6%)
**Very bad**	262 (1.5%)	116 (3.3%)	146 (1.1%)	714 (0.1%)	315 (0.1%)	399 (0.1%)	136 (1.8%)	48 (2.9%)	88 (1.5%)	986 (0.2%)	476 (0.2%)	510 (0.2%)

For peer review only

For peer review only - http://bmjopen.bmj.com/site/about/guidelines.xhtml

Table 4. Odds ratio of autism, age, and gender in predicting poor health* in the whole population

Variable		Odds ratio	95% confidence interval
Autism	No autism (reference)	-	
	Autism	11.339	10.983-11.707
Age	0-15 (reference)	-	
	16-24	2.137	2.098-2.176
Gender	Male (reference)	-	
	Female	1.126	1.106-1.147
Constant		.020	

*fair, bad or very bad health

Table 5. Odds ratios of age and gender in predicting poor health* in the population with autism

Variable		Odds ratio	95% confidence interval
Age	0-15 (reference)	-	
	16-24	1.206	1.133-1.284
Gender	Male (reference)	-	
	Female	1.635	1.527-1.750
Constant		.252	

*fair, bad or very bad health

For peer review only - http://bmjopen.bmj.com/site/about/guidelines.xhtml

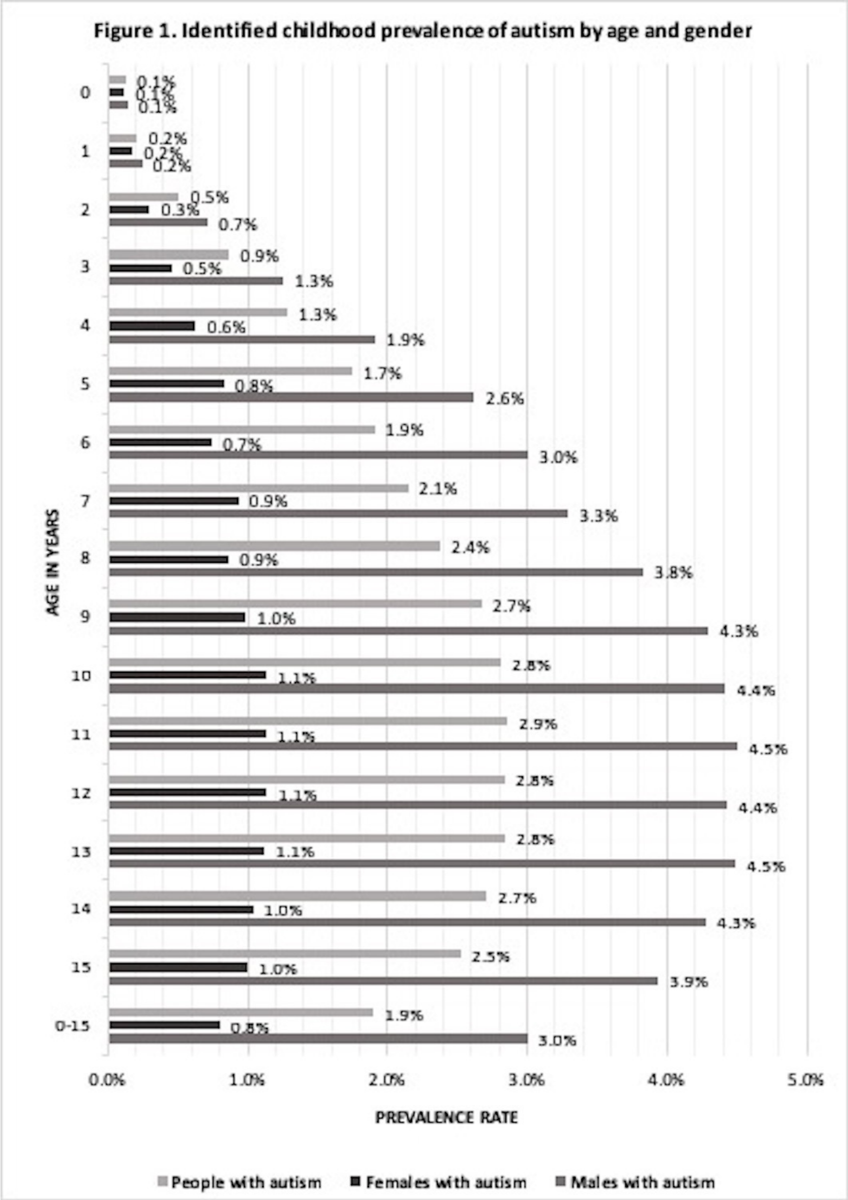

Figure 1. Identified childhood prevalence of autism by age and gender

90x127mm (300 x 300 DPI)

STROBE 2007 (v4) Statement—Checklist of items that should be included in reports of *cross-sectional studies*

Section/Topic	Item #	Recommendation	Reported on page #
**Title and abstract**	1	(*a*) Indicate the study's design with a commonly used term in the title or the abstract	Page 1
		(*b*) Provide in the abstract an informative and balanced summary of what was done and what was found	Page 2
**Introduction**			
Background/rationale	2	Explain the scientific background and rationale for the investigation being reported	Page 4-5 Section: Introduction
Objectives	3	State specific objectives, including any prespecified hypotheses	Page 5 Section: Introduction
**Methods**			
Study design	4	Present key elements of study design early in the paper	Page 5-8 Sections: Methods/Procedures, Data source, Census variables
Setting	5	Describe the setting, locations, and relevant dates, including periods of recruitment, exposure, follow-up, and data collection	Page 5-6 Sections: Methods/Procedures, Data source
Participants	6	(*a*) Give the eligibility criteria, and the sources and methods of selection of participants	Page 6-8 Sections: Methods/Data source, Census variables
Variables	7	Clearly define all outcomes, exposures, predictors, potential confounders, and effect modifiers. Give diagnostic criteria, if applicable	Page 6-8 Section: Methods/Census variables
Data sources/ measurement	8*	For each variable of interest, give sources of data and details of methods of assessment (measurement). Describe comparability of assessment methods if there is more than one group	Page 6-8 Section: Methods/Census variables
Bias	9	Describe any efforts to address potential sources of bias	Page 5-8

For peer review only - http://bmjopen.bmj.com/site/about/guidelines.xhtml

			Section: Methods
Study size	10	Explain how the study size was arrived at	Page 5-9<br>Sections: Methods/Data source, Census variables
Quantitative variables	11	Explain how quantitative variables were handled in the analyses. If applicable, describe which groupings were chosen and why	Page 8<br>Section: Methods/Data analysis
Statistical methods	12	(*a*) Describe all statistical methods, including those used to control for confounding	Page 8<br>Section: Methods/Data analysis
		(*b*) Describe any methods used to examine subgroups and interactions	Page 8<br>Section: Methods/Data analysis
		(*c*) Explain how missing data were addressed	Page 6-8<br>Sections: Methods/Data source, Census variables
		(*d*) If applicable, describe analytical methods taking account of sampling strategy	N/A
		(*e*) Describe any sensitivity analyses	N/A
**Results**			
Participants	13*	(a) Report numbers of individuals at each stage of study—eg numbers potentially eligible, examined for eligibility, confirmed eligible, included in the study, completing follow-up, and analysed	Page 9<br>Section: Results/Number (%) of children and young people with autism by age and gender<br>Page 18 Table 2<br>Figure 1
		(b) Give reasons for non-participation at each stage	N/A
		(c) Consider use of a flow diagram	N/A
Descriptive data	14*	(a) Give characteristics of study participants (eg demographic, clinical, social) and information on exposures and potential confounders	Page 9<br>Section: Results/Number (%) of children and young

For peer review only

For peer review only - http://bmjopen.bmj.com/site/about/guidelines.xhtml

			people with autism by age and gender<br>Page 18 Table 2<br>Figure 1
		(b) Indicate number of participants with missing data for each variable of interest	Page 6-8<br>Sections: Methods/ Census variables
Outcome data	15*	Report numbers of outcome events or summary measures	N/A
Main results	16	(*a*) Give unadjusted estimates and, if applicable, confounder-adjusted estimates and their precision (e.g., 95% confidence interval). Make clear which confounders were adjusted for and why they were included	Page 9-10<br>Section: Results/General health<br>Page 18 Table 3<br>Page 19 Tables 4 and 5
		(*b*) Report category boundaries when continuous variables were categorized	Page 8<br>Section: Methods/Data analysis<br>Page 18 Table 2<br>Pages 19 Table 3<br>Page 20 Tables 4 and 5<br>Figure 1
		(*c*) If relevant, consider translating estimates of relative risk into absolute risk for a meaningful time period	N/A
Other analyses	17	Report other analyses done—eg analyses of subgroups and interactions, and sensitivity analyses	N/A
**Discussion**			
Key results	18	Summarise key results with reference to study objectives	Page 10-12<br>Section: Discussion/ Principal findings and interpretation, Comparison with existing literature
Limitations	19	Discuss limitations of the study, taking into account sources of potential bias or imprecision. Discuss both direction and magnitude of any potential bias	Page 12-13<br>Section: Strengths and

For peer review only - http://bmjopen.bmj.com/site/about/guidelines.xhtml

			limitations
Interpretation	20	Give a cautious overall interpretation of results considering objectives, limitations, multiplicity of analyses, results from similar studies, and other relevant evidence	Page 10-12<br>Section: Discussion/ Principal findings and interpretation, Comparison with existing literature
Generalisability	21	Discuss the generalisability (external validity) of the study results	Page 13<br>Section: Implications for clinicians
**Other information**			
Funding	22	Give the source of funding and the role of the funders for the present study and, if applicable, for the original study on which the present article is based	Page 14<br>Section: Funding

*Give information separately for exposed and unexposed groups.

Note: An Explanation and Elaboration article discusses each checklist item and gives methodological background and published examples of transparent reporting. The STROBE checklist is best used in conjunction with this article (freely available on the Web sites of PLoS Medicine at http://www.plosmedicine.org/, Annals of Internal Medicine at http://www.annals.org/, and Epidemiology at http://www.epidem.com/). Information on the STROBE Initiative is available at www.strobe-statement.org.

For peer review only
